# Purpura Hemorrhagica in the Bovine Species, with Report of a Case1Paper read before the Missouri Valley Veterinary Association, December 13, 1895.

**Published:** 1896-03

**Authors:** G. T. Netherton

**Affiliations:** Gallatin, Mo.


### PURPURA HEMORRHAGICA IN THE BOVINE SPECIES, WITH REPORT OF A CASE.[1]

BY G. T. NETHERTON, M.D.C.,
GALLATIN, MO.

PURPURA hemorrhagica is a disease frequently met with in our practice, and is so well understood by the profession that I deem it unnecessary to enter into a minute pathological description in this brief paper; suffice it to say, that it is a disease due to a deterioration of the blood, with impaired function of the vasomotor nerves governing the smaller bloodvessels and capillaries, the red blood-corpuscles having a contracted appearance, while the white corpuscles seem to increase in number; also the aqueous portion of the blood is increased and the fibrinous portion is decreased, thus lessening the coagulability of the

[1] Paper read before the Missouri Valley Veterinary Association, December 13, 1895.

blood. The general appearance of the blood is similar to fresh blood having been treated with ammonia. Purpura, as a general rule, is said to be a sequel to some other debilitating disease; but our experience has taught us that there are many exceptions to this rule, as we have been called to treat cases in fairly good flesh, running on pasture, with no history of any previous trouble.

All of our text-books, together with all reliable veterinary literature that I have had the privilege of reading, confine the disease of purpura hemorrhagica to the equidæ or soliped, and my own practical experience confirms the position of our text-books with one exception, viz.:

August 19, 1895, I was called to see a Jersey cow, in full flow of milk; calf three and a half months old; cow in good flesh and on good pasture, with drinking water supplied from a well, with no history of previous sickness.

*Semiology.* Lacteal secretions largely suppressed; large, patchy, circumscribed swelling on inside of thighs, as well as on back and loins; head and ears swollen; ears hanging like heavy pads to the sides of the head; the eyelids swollen shut, with œdema of the conjunctiva; the udder and teats swollen and œdematous, giving it more the appearance of some wild animal than that of a neat Jersey cow, which she was when in her normal condition. Pulse and temperature slightly accelerated. The owner being an intelligent gentleman, the editor of a county newspaper, asked for my diagnosis. My reply was that if I had these symptoms in a horse I would call it purpura hemorrhagica, but as I have never read nor witnessed a case like this in the cow, I should not name it, but would administer a saline cathartic and see her again the next day, providing she lived that long.

Upon my visit next day I found the characteristic exudation of bloody serum standing out profusely on the diseased surface I then remarked to the owner that we had a typical case of purpura hemorrhagica in the bovine species. There developed considerable exfoliation about the head and eyes and upon the udder and teats. The disease yielded nicely to the ordinary treatment for purpura in the soliped, and the cow made a good recovery.

